# Sensitivity, specificity and prevalences**—**comparison of four screening tests for temporomandibular disorders

**DOI:** 10.22514/jofph.2025.069

**Published:** 2025-12-12

**Authors:** Maria Ertl, Florian Beuer, Axel Bumann

**Affiliations:** ^1^CMD-Zentrum Berlin, 13187 Berlin, BE, Germany; ^2^Department of Prosthodontics, Geriatric Dentistry and Craniomandibular Disorders, Charité Universitätsmedizin Berlin, 10117 Berlin, BE, Germany; ^3^MESANTIS 3D DENTAL-RADIOLOGICUM, Bumann-Academy, 13187 Berlin, BE, Germany

**Keywords:** Temporomandibular disorder, Screening, Sensitivity, Specificity

## Abstract

**Background**: This prospective clinical study investigates whether functionally compensated but clinically inapparent temporomandibular disorder (TMD) findings can be detected in individuals without pre-existing medical conditions. **Methods**: A total of 200 participants (10–50 years) without a medical history were examined using screening methods: Craniomandibular Disorders (CMD)-Short Finding, CMD-Screening, Preventive Manual Structural Analysis (PMSA), and Preventive Structural Stress Screening (PSSS). The Diagnostic Criteria for Temporomandibular Disorders (DC/TMD) Axis I served as the reference standard. Five diagnoses were analyzed based on prevalence, sensitivity, specificity, predictive values, 95% confidence intervals, and *p*-values (McNemar, Cochran Q). **Results**: TMD findings were detectable. PMSA and PSSS always produced identical results (*p* = 1.000), differing significantly from CMD-Short Finding for arthralgia and disc displacement (*p* < 0.001), and from CMD-Screening for myogenic pain (*p* < 0.001). No significant differences were found between PMSA/PSSS and CMD-Screening for arthralgia (*p* = 0.528) or CMD-Short Finding for myogenic pain (*p* = 0.490). Not all tests achieved a 70% sensitivity and a 95% specificity. The most important results are summarized here: arthralgia (0.0%–15.0%) by PMSA/PSSS (sensitivity 96.00% [79.65–99.90], specificity 96.57% [92.69–98.73], *p* = 0.125); myogenic pain (34.5%–49.5%) by CMD-Screening (excluding digastric muscle: sensitivity 84.15% [74.42–91.28], specificity 100.00% [96.92–100.00], *p* < 0.001) and by PMSA/PSSS (including digastric muscle: sensitivity 98.02% [93.03–99.76], specificity 100.00% [96.34–100.00], *p* = 0.500); disc displacement (14.5%–20.5%) by CMD-Short Finding (sensitivity 100.00% [85.18–100.00], specificity 96.61% [92.77–98.75], *p* = 0.031); degenerative joint disease and disc displacement without reduction with limited opening showed very low prevalence (0.0%–1.0%), which results in limiting reliability. **Conclusions**: TMD findings were detectable in asymptomatic individuals. PMSA and PSSS were the most suitable screening tools. The results support the need for regular screening, and further studies. **Clinical Trial Registration**: DRKS00035175, https://drks.de/search/en/trial/DRKS00035175, retrospectively registered.

## 1. Introduction

Temporomandibular disorders (TMD) cover functional disorders of the temporomandibular joints and masticatory muscles, often associated with pain, joint sounds, and restricted mouth opening (RMO) [1]. TMD affects a considerable proportion of the population. For example, the American study “Orofacial Pain: Prospective Evaluation and Risk Assessment” found an annual incidence of 3.9% [2].

Compensated findings refer to adaptive tissue responses triggered by environmental factors, which result in compensation without subjective symptoms reported by the patient. Conversely, clinical examination has been demonstrated to yield reproducible, provocative latent findings [3]. The development of such compensated findings depends on both the intensity and duration of environmental factors and the body’s ability to adapt and compensate. These findings are accompanied by destructive changes in shape or pain, which are already apparent in the medical history, *e.g.*, indication of pain or cracking/grating noises [3].

Case law in Germany requires the treating (specialist) dentist to diagnose functional disorders and hidden findings before treatment [4, 5]. Both the German Society of Dentistry and Oral Medicine [6] and Section 630e of the Patient Rights Act [7], require a functional examination of the craniomandibular system prior to dental treatment in order to identify such compensated findings.

A screening test is diagnostic tool used to assess the probability of disease in an asymptomatic individuals [8]. Likewise, screening tests are used in human medicine for the early detection of kidney dysfunction (*e.g.*, in diabetes or hypertension [9]), prostate cancer [10], or cardiovascular disease [11]. Screening procedures are also well established in dentistry. The most commonly used is the Periodontal Screening Index [12], which is routinely conducted every two years in general dental practice [13]. In Germany, several screening methods have been implemented for the early detection of temporomandibular disorders (TMD) in asymptomatic individuals. These include the craniomandibular disorder (CMD)-Screening developed by the German Society of Craniomandibular Function and Disorders in cooperation with the German Society of Dentistry and Oral Medicine (CMD-Screening) [14], CMD-Short Finding [15, 16, 17, 18], Preventive Manual Structural Analysis (PMSA) [19] and Preventive Structural Stress Screening (PSSS) [19, 20].

Clinical, instrumental, and manual functional analyses are recognized as scientifically valid methods for examining TMD [6]. However, it remains unclear which of these four TMD screening tools is most effective in identifying compensated findings—that is, findings that are present but not evident in the patient’s medical history, (according to Bumann *et al*. [3]).

The Diagnostic Criteria for Temporomandibular Disorders (DC/TMD), first published in 2014 is an internationally recognized examination method of the stomatognathic system, based on the Research Diagnostic Criteria for Temporomandibular Disorders (RDC/TMD) [21] and translated into German [22]. Due to its reliability and validity, it is recommended for scientific work and diagnosing symptomatic patients [21, 23, 24]. Nonetheless, recent discussions have questioned its status as the definitive gold standard for scientific investigations [25, 26].

This study addresses whether TMD findings can be present despite the absence of symptoms in the patient’s medical history. Such findings are described in this study as *compensated findings*. In addition, a comparison of the four TMD screening tests is conducted to assess which test most effectively detects compensated findings. The DC/TMD Clinical Examination Protocol was chosen as the reference standard for this comparison. The following hypotheses were tested: (A) The screening tests differ in their diagnostic quality. (B) The screening tests differ from the reference standard. (C) The screening tests differ in terms of prevalence.

## 2. Materials and methods

### 2.1 Subjects

Between December 2021 to November 2022, a prospective cross-sectional study was conducted in Berlin as part of a dissertation [27]. The study was approved by the Ethical Committee (EA2/213/21). All subjects provided signed informed consent for participation in this research. The study was conducted according to the World Medical Association Declaration of Helsinki.

New patients and their family members, ages 10 to 50 years, with no history of TMD symptoms, were recruited in a consecutive series and examined at a private dental office (A. Bumann, Berlin). Participants aged 10 to 18 years represented orthodontic patients, while those aged 19 to 50 years were seen prior to conservative or prosthetic dental treatment. The lower age limit of 10 years was chosen to ensure that underage participants were likely undergoing orthodontic treatment, which in Germany (typically) begins during the second transitional dentition phase. For the general dental target group, relatives of the underage patients were recruited. These were usually their legal guardians, so an upper age limit of 50 years seemed feasible. Participants were recruited consecutively, with nearly 100 individuals in each age group (10–18 and 19–50 years), and with approximately equal gender distribution in both groups. The subdivision into age groups was used for some dissertation hypotheses, but the subdivisions are irrelevant to the hypotheses in this study (Fig. 1).

**Fig. 1.** Graphical abstract. Created by Maria Ertl. A total of 223 participants were recruited, and 23 subjects were excluded. A total of 200 Participants were tested by four TMD screening tests and DC/TMD (reference standard). The following hypotheses were tested: (A) The screening tests differ in their diagnostic quality (target of a 70% sensitivity, a 95% specificity), (B) The screening tests differ from the reference standard (McNemar test), and (C) The screening tests differ in terms of prevalence (Cochran Q test, McNemar test). Results: TMD findings were detectable in asymptomatic individuals; TMD screening tools are necessary; DJD and DD w/o R showed very low prevalence, which yields in limiting reliability; PMSA and PSSS were the most suitable screening tools. DD: disc displacement with reduction; DD w/o R: disc displacement without reduction with limited opening; DJD: degenerative joint disease; compensate: TMD findings can be present despite the absence of symptoms in the patient’s medical history; PMSA: Preventive Manual Structural Analysis; PSSS: Preventive Structural Stress Screening; CMD: Craniomandibular Disorders; DC/TMD: Diagnostic Criteria for Temporomandibular Disorders.

Subjects were included if asymptomatic for TMD symptoms for the previous three months and aged 10 to 50 years. Exclusion criteria were: head/face/jaw joint pain, facial tension, cracking, grinding, mouth-opening limitation, or mouth-closing blockage for the previous three months; systemic rheumatic, neurological, neuropathic, collagen, vascular, and autoimmune diseases; fibromyalgia; depression; pregnancy; head/neck trauma within the previous two months; medication use within the previous six weeks; use of analgesics, narcotics, and muscle relaxants within the previous two weeks; conservative or orthodontic treatment in the previous three months; radiotherapy to the head and neck; or temporomandibular joint surgery. In the context of the German legal system, individuals who self-report as symptom-free are of forensic interest. Therefore, this study only included participants with no history of symptoms against which the TMD screening tests could be compared. In addition, TMD has a smooth transition from a healthy to a diseased state, so a control group with symptoms was deliberately omitted (Fig. 1).

### 2.2 Procedure

The following materials were required for the procedure: ruler, scales, blue and red occlusion foil, grease pencil, stopwatch, four cotton rolls, findings sheets for the PMSA, PSSS, CMD-Short Finding, CMD-Screening, DC/TMD, DC/TMD Symptom Questionnaire, and the Study-Specific Dental TMD Screening Questionnaire.

The doctoral student was responsible for all aspects of the recruitment, examination, and data analysis processes. The participants were anonymized before the data was evaluated. All participants completed the aforementioned questionnaires and the informed consent form in advance at home and brought these with them on the day of the study.

The four screenings were conducted for each subject in alternating order and on the same examination day. The DC/TMD Clinical Examination Protocol served as the reference standard and was also conducted on the same examination day following the screening tests. For the duration of the five tests, each participant was given one hour (Fig. 1).

Calibration was ensured by the following measures: Metric measurements were taken to the nearest millimeter using a ruler. Furthermore, only reproducible findings were evaluated and active movements were measured after three repetitions. Before each palpation, the palpation pressure was calibrated using a scale.

#### 2.2.1 Reference standard DC/TMD Axis I

For the purposes of this study, only Axis I, as measured by the DC/TMD Symptom Questionnaire and the DC/TMD Clinical Examination Protocol was considered. The DC/TMD procedure is described below based on the discussions of Schiffman and Ohrbach *et al*. [22].

In general, the right and left sides of the body were examined separately for corresponding findings.

The DC/TMD Symptom Questionnaire was utilized to document acute or past facial pain and discomfort experienced during chewing or opening the mouth. These symptoms were meticulously recorded in the medical history and assigned to one side, if feasible. Given the absence of a history of symptoms, it was not possible to localize any acute pain in the DC/TMD Examination Form.

Initially, the overjet, the overbite, and any midline shifts were measured with millimetric precision using a ruler. The mouth opening movement was described as straight, deviation or deflection.

The pain-free mouth opening, maximum active mouth opening, maximum passive mouth opening, laterotrusion right/left, and protrusion were then measured using a ruler. If the maximum assisted mouth opening had to be aborted, this was noted. Pain that occurred during active movements was differentiated according to its location (temporalis muscle/masseter muscle/joint/other masticatory muscles/non-chewing muscles) and quality (Pain/Familiar Pain). With regard to the temporalis muscle, pain could be differentiated into Pain, Familiar Pain, and Familiar Headache.

Active movements (mouth opening, mouth closing, lateral movements, and protrusion) were examined for joint noises (grinding and cracking). These sounds were classified according to whether they were perceived by the patient or the examiner. If cracking noises occurred together with pain, it was differentiated according to whether they were associated with Pain or Familiar Pain. If lockjaw occurred during mouth opening or wide opening position, this was noted, and a distinction was made as to whether the patient or the examiner was able to release it.

The temporalis muscle, with its associated temporalis tendon, the masseter muscle, the digastric venter anterior/posterior muscle, and the lateral pterygoid muscle, were each palpated in their entirety. The temporomandibular joints were palpated bilaterally and around the lateral condylar pole. The palpation pressure for the temporalis muscle, the masseter muscle, and for the region around the lateral condylar pole was 1 kg in each case. Palpation of the lateral pole of the condyle, the retro- and submandibular region, the temporalis tendon, and the lateral pterygoid muscle was performed with a pressure of 0.5 kg. Generally pain on palpation was categorized into: Pain, Familiar Pain, and Referent Pain, with the exception of palpation onto the temporal muscle with an added category of Familiar Headache [22].

#### 2.2.2 CMD-Short Finding

The CMD-Short Finding procedure is briefly described based on the publications of Ahlers *et al*. [16, 17, 18] and the methods description partly reproduces their wording. A maximum of six non-side-specific parameters could be selected. A deviation or deflection greater than 2 mm was counted as a mouth-opening deviation. An active mouth opening of less than 38 mm was counted as a restriction of the mouth opening. The temporomandibular joints were examined for clicking or grinding noise when opening or closing the mouth. If the habitual occlusion made a dull or intermittent noise, this was counted as a positive finding. A non-physiological dynamic occlusion was present if occlusal abrasion, grinding facets, or balance/hyper-balance contacts that were not age-appropriate were present. Palpation of the masseter muscle pars superficialis, the temporalis muscle pars anterior, and the digastric muscle posterior belly were performed with a pressure of 0.5 kg [18]. In the CMD-Short Finding, the reliability of recognizing temporomandibular joint disorders was increased by summing the individual parameters [15, 16, 17, 18]. To maintain comparability across the TMD screening tests, parameters summation was not performed in this study. Further clinical functional analysis is recommended when at least two parameters are positive [16, 17, 18] (Fig. 2, Ref. [16, 17, 18]).

**Fig. 2.** Flow chart of the Craniomandibular Disorders Short Finding. Created by Maria Ertl based on the template in Ahlers *et al*.’s [16, 17, 18] publications. A checkmark is placed if a finding applies. All positive parameters are totaled. A clinical functional analysis is recommended for further diagnostics in cases with at least two positive parameters. No summation of parameters was performed in this study to ensure the comparability of the TMD screening tests. CMD: craniomandibular disorders.

#### 2.2.3 CMD-Screening

The CMD-Screening procedure is briefly described based on a discussion by the German Society of Craniomandibular Function and Disorders and the methods description partly reproduces their wording [14]. Anamnestic questions and examination parameters were answered and noted on the findings form in a nonspecific manner. The survey asked about acute pain once or more per week in the temporomandibular joint in the temporal/facial/jaw area and about pain when opening the mouth or chewing. The questionnaire also asked about blockages or difficulties once or more per week when opening the mouth. Following the DC/TMD guidelines, the masticatory muscles were examined for pain, and the temporomandibular joints were assessed for both noise and pain. Palpation of the masseter muscle pars superficialis and the temporalis muscle pars anterior/media/posterior was performed with a pressure of 1 kg. An active mouth opening of less than 40 mm was counted as an RMO. Disorders of habitual occlusion were determined acoustically and visually with Shimstock foil. The temporomandibular joints were tested for clicking or grinding noise during mouth opening, mouth closing, protrusion and laterotrusion. The nine possible parameters were color-coded. In the medical history questions, pain in the temporomandibular joints and masticatory muscles and the RMO score were marked red, and occlusal disorders or temporomandibular joint noises were marked yellow [14]. This study did not use color coding to compare the TMD screening tests. Extended diagnostics are recommended for a yellow parameter and considered urgently indicated for a red one (Fig. 3, Ref. [14]).

**Fig. 3.** Flow chart of the Craniomandibular Disorders Screening. Created by Maria Ertl based on the template of the German Society of Craniomandibular Function and Disorders in the German Society of Dentistry and Oral Medicine (CMD-Screening) [14]. Extended diagnostics are recommended for a yellow parameter and considered urgently indicated for a red one [14]. This study did not use color coding to compare the TMD screening tests. CMD: craniomandibular disorders; DC/TMD: Diagnostic Criteria for Temporomandibular Disorders.

#### 2.2.4 Preventive Manual Structural Analysis

The PMSA procedure is summarized according to the publications of Bumann *et al*. [19], with parts of the description adapted from the original wording. A side-specific examination of the patients was conducted. Pain, end feel, and muscle strength were assessed qualitatively. Active and passive mouth opening, lateral movements, protrusion, and retrusion were measured and analyzed for pain. The end feel was determined for pain-free passive mouth opening. If pain occurred, it was assigned to the masticatory muscle or temporomandibular joints by localization. Condylar mobility was described during mouth opening and protrusion. An active mouth opening of less than 43 mm was counted as an RMO. The temporomandibular joints were examined for crepitus and pain using dynamic compression and assessed for clicking noise using dynamic translation. The bilaminar zone was tested for pain using passive compression in the cranial, dorsal, dorsocranial, dorsolateral, and dorsolateral-cranial directions. The joint capsule was assessed for pain and mobility using ventral translation and caudal traction. Palpation of the masseter muscle pars superficialis/profunda and the temporalis muscle pars anterior/media/posterior was performed with a pressure of 1 kg, whereas for the digastric muscle anterior/posterior bellies it was only 0.5 kg. The isometric tests lasted 80 seconds each and were carried out for mouth opener muscles and mouth closer muscles. The suprahyoidal structures were tested for sagittal and vertical restrictions. Furthermore, static and dynamic occlusion, bruxism, and dysfunctions were analyzed concerning their influence on the existing findings. Finally, suspected diagnoses related to stress and restriction were summarized [19]. Physiological findings are highlighted in green, and compensated findings are highlighted in yellow [19]. The present study did not include end feel, occlusal vectors, pain quality, restrictions, condylar mobility, isometric tests, or the use of color coding because the objective was to compare TMD screening tests (Fig. 4, Ref. [19]).

**Fig. 4.** Flow chart of the Preventive Manual Structural Analysis. Created by Maria Ertl based on the template in the publication of Bumann *et al*. [19]. The red outline indicates parameters that can be assigned to the DC/TMD diagnoses. The other parameters were evaluated as additional information in this study, with no distinction being made between the quality of the pain and muscle strength and only whether the respective parameter was present.

#### 2.2.5 Preventive Structural Stress Screening

The PSSS procedure is briefly described based on the publications of Bumann *et al*. [19, 20] and the methods description partly reproduces their wording. Up to six non-side-specific, color-coded parameters could be selected. In addition, neither the quality of pain nor the final Sensation was specified. The passive mouth opening was examined for pain in the masticatory muscles or temporomandibular joint. The active mouth opening was measured metrically and assessed for asymmetries, defined as deviation or deflection exceeding 2 mm. An active mouth opening of less than 43 mm was counted as an RMO. The temporomandibular joints were examined for crepitus using dynamic compression and for cracking noise using dynamic translation. Passive compression was performed in the dorsolateral and dorsolateral-cranial directions to assess the bilaminar zone. Palpation of the masseter muscle pars superficialis/profunda and the temporalis muscle pars anterior/media/posterior was performed with a pressure of 1 kg. The anterior and posterior bellies of the digastric muscle were palpated with a pressure of 0.5 kg. The dentition was examined for signs of attrition and for the presence of balance or hyperbalance contact. This study did not assign the parameters to a green, yellow, or red result field because they were examined individually. If two green parameters or one green and one yellow parameter are present, this results in a yellow outcome field. Two yellow parameters—or one yellow combined with one green—lead to a red outcome field. A yellow result suggests that further diagnostics are recommended, and if the result field is red, diagnostics are urgently indicated (Fig. 5, Ref. [19, 20]).

**Fig. 5.** Flow chart of the Preventive Structural Stress Screening. Created by Maria Ertl based on the template in the publications of Bumann *et al*. [19, 20]. Two green or the combination of a green and a yellow parameter can lead to a yellow result field. The presence of two yellow parameters or one yellow and one green parameter results in a red result field. If the result field is yellow, extended diagnostics are recommended, and if the result field is red, diagnostics are urgently indicated [19, 20]. This study did not assign the parameters to a green, yellow, or red result field because they were examined individually. DL: dorsolateral; DLC: dorsolateral-cranial.

#### 2.2.6 Comparability of the screening tests

Five of the twelve DC/TMD diagnoses [22] were used in this study: myogenic pain, arthralgia, disc displacement (DD) with reduction, DD without reduction with limited opening (DD w/o R), and degenerative joint disease (DJD). Due to the absence of imaging and reported symptoms in the participants’ medical history, several modifications were made: (local) myalgia, myofascial pain with or without reduction, and TMD headache were combined under the term *myogenic pain*. For DD with reduction, intermittent jaw clenching was not included. In addition, DD w/o R without RMO was not included.

Since the screening tests vary significantly in their parameters, scale levels, and evaluation methods. The screening test parameters were assigned to each of the five DC/TMD diagnoses to compare the four TMD screening tests. A positive screening parameter was interpreted as evidence for the corresponding diagnosis (Fig. 6).

**Fig. 6.** Flow chart of the specific parameters of the four screening tests and for the five diagnoses. Created by Maria Ertl. Positive parameters were taken to indicate the presence of the diagnosis in question. PMSA: Preventive Manual Structural Analysis; PSSS: Preventive Structural Stress Screening; CMD: craniomandibular disorders; D: dorsal; DC: dorsocranial; DL: dorsolateral; DLC: dorsolateral-cranial; C: cranial; DD: disc displacement with reduction; DD w/o R: disc displacement without reduction with limited opening; DJD: degenerative joint disease.

#### 2.2.7 Adapted DC/TMD diagnostic algorithm

DC/TMD diagnostic algorithm was modified for arthralgia, myogenic pain, and DJD regarding the required positive history [22], as the patients in this study had no history of symptoms.

In routine clinical practice, a significant proportion of patients exhibit no symptoms but demonstrate compensated findings during screening procedures. In order to ensure a realistic reflection of the condition, the study exclusively included patients who had not exhibited any symptoms. As the DC/TMD diagnostic algorithms for arthralgia, myogenic pain, and degenerative joint disease require symptoms, these were adjusted accordingly. For better differentiation of these three DC/TMD diagnoses, results were presented using modified and unmodified DC/TMD diagnostic algorithms. The results of unmodified diagnostic algorithms were described as *decompensated diagnoses* (*e.g.*, decompensated arthralgia). The results of modified diagnostic algorithms were called *compensated diagnoses* (*e.g.*, compensated arthralgia).

The flowchart of the five DC/TMD diagnoses, partially adapted for this study based on the discussion by Schiffman and Ohrbach *et al*. [22] is briefly described below:

Using the DC/TMD diagnostic algorithm, decompensated arthralgia could only be diagnosed if the patient had a positive pain history which was confirmed by the examiner [22]. Furthermore, only Familiar Pain during mouth opening, excursive movements, or palpation of the temporomandibular joint were considered [22]. In contrast, for compensated arthralgia findings, neither a pain history nor confirmation during examination was required; instead, unfamiliar (unknown) pain in the temporomandibular joint was included (Fig. 7, Ref. [22]).

**Fig. 7.** Flow chart of the DC/TMD diagnostic algorithm for arthralgia, myogenic pain, disc displacement with reduction, disc displacement without reduction with limited opening, and degenerative joint disease. Created by Maria Ertl based on the template of the German DC/TMD protocol of the International RDC/TMD Consortium Network [22] references to the DC/TMD Examination Form [E] and the DC/TMD Symptom Questionnaire [SQ] are in the square brackets. TMD: temporomandibular disorders; TMJ: temporomandibular joint; CT: computer tomography; MRI: magnetic resonance imaging.

Using the DC/TMD diagnostic algorithm, decompensated muscle findings could only be diagnosed if the patient had a positive pain history which was confirmed by the examiner [22]. Furthermore, only Familiar Pain during mouth opening or palpating the masseter/temporalis muscles were considered [22]. In contrast, for compensated muscle findings, both the patient’s pain history and its confirmation were disregarded. Any unfamiliar pain in the masticatory muscles (temporalis muscle, masseter muscle) was counted regardless of its distribution and pain transmission during mouth opening and palpation. Additionally, muscle findings were evaluated both with and without including pain on palpation of the digastric muscle. In the DC/TMD examination procedure, the retro- and submandibular structures, including the digastric muscle, are palpated. However, they are not included in the diagnostic algorithm for muscle pain. Reporting results both with and without the digastric muscle prevents loss of information and helps avoid misinterpretation. For example, it ensures that the screening tests are not misinterpreted as being inadequate for recording the mouth closers. For this reason, the results for myogenic pain are presented both ways (submandibular region, posterior mandibular region) (Fig. 7).

According to the diagnostic algorithm, DJD could be diagnosed in the absence of current temporomandibular joint (TMJ) noises by history, but examiners had to confirm the presence of crepitus [22]. For compensated DJD, the grinding noise perceived by the subjects was counted, regardless of their medical confirmation (Fig. 7).

No distinction was made between compensated and decompensated for DD and DD w/o R. A cracking noise was classified as DD if no previous cases of lockjaw were reported in the DC/TMD Symptom Questionnaire and if the noise was also perceived by the patient during the examination. Moreover, the examiner was required to DC/TMD Decision Tree identify any clicking noises observed during mouth opening and closing, mouth opening and exclusive movement, or mouth closing and exclusive movement. Both DD with reduction with intermitted locking and DD without reduction without RMO were not taken into consideration (Fig. 7).

In the event that a previous case of lockjaw was confirmed in the DC/TMD Symptom Questionnaire and a maximum assisted mouth opening of 40 mm or less was observed during the examination, the resulting DC/TMD diagnosis was DD w/o R. When taking the second part of the DC/TMD (if “No” at 9 and 10 in Symptom Questionnaire), no DD w/o R could be diagnosed. Questions 11 and 12 of the Symptom Questionnaire addressed current jaw lock episodes. A “Yes” response to either question led to exclusion from the study (Fig. 7).

The examination quality was ensured via eight years of professional experience and by completing the TMD curriculum [28] to learn the manual examination techniques of the PMSA and PSSS. The examination techniques of the CMD-Short Finding [16, 17, 18], CMD-Screening [14], and DC/TMD [21, 22] were learned by studying the literature and using the instruction video from the University of Leipzig [23]. Rauch *et al*. [23] demonstrated that DC/TMD can be mastered and applied without errors during self-study using this instructional video. The German assessment instruments were used to calibrate the examiner for performing the DC/TMD [22].

#### 2.2.8 Statistical analysis

The target sample size of 192 to 216 subjects was confirmed by the Institute of Biometry and Clinical Epidemiology at Campus Charité Mitte. The number was determined using the function power.diagnostic.test from the R package MKmisc, assuming that ten examinations/day, two days per month, could be performed within a year as a part of the dissertation [27]. The power was set at 80%.

The prospective design ensured complete data collection; therefore, missing data were not an issue. The statistical analysis for age distribution was performed using the Shapiro and MannWhitney U tests. The scale levels of the parameters were nominal. Prevalence values of the five DC/TMD diagnoses were given for each screening. Differences were analyzed using the Cochran Q test. If a significant difference was found, pairwise comparisons were performed using the McNemar test. Sensitivity, specificity, positive predictive value (PPV), and negative predictive value (NPV) were calculated using a four-field table.

The PPV and NPV were each assigned with study prevalence and with age-specific weighted [29] reference prevalence [1, 30, 31, 32, 33]. This study demonstrated, among other findings, that binary test outcomes are strongly influenced by the underlying prevalence.

The 95% confidence intervals (CI) and value verifications were calculated using the MedCalc Software Ltd diagnostic test evaluation calculator v.20.112 (https://www.medcalc.org/calc/diagnostic_test.php). In the literature, a 70% sensitivity and a 95% specificity are acceptable values for TMD tests [34]; therefore, this study followed these guidelines. The described values were reported for each DC/TMD diagnosis.

Finally, statistically significant differences between DC/TMD and the respective TMD screening test were analyzed using McNemar tests. In addition, a side-specific comparison was made between the PMSA and DC/TMD, considering the right/left correlation using a general estimating equation and odds ratio (OR). The significance level was two-tailed and set at α = 0.05.

## 3. Results

A total of 223 participants were recruited between December 2021 and November 2022, of whom 200 were (ultimately) included in the study. Seventeen subjects missed their appointments or were no longer available. Six individuals were excluded prior to participation due to self-reported headaches or clicking noises within the previous three months. The following figure shows the study flow diagram (Fig. 8).

**Fig. 8.** Study flow diagram. Created by Maria Ertl. New patients and their family members were recruited in a consecutive series. Subjects were included if asymptomatic for TMD symptoms for the previous three months and aged 10 to 50 years. The four screenings were conducted for each subject in alternating order and on the same examination day. The DC/TMD Axis I served as the reference standard and was also conducted on the same examination day following the 4 screening tests. DD: disc displacement; DD w/o R with RMO: disc displacement without reduction with restricted mouth opening; CMD: craniomandibular disorders; TP: true positive; TN: true negative; FP: false positive; FN: false negative.

Of the 200 patients aged between 10 and 50 who participated in the study, 98 (49.0%) were male and 102 (51.0%) were female. The mean age was 22.85 ± 11.26 years. The age distribution deviated significantly from normality in both groups (male *p* < 0.001; female *p* < 0.001). The female subjects (mean age = 24.42, standard deviation = 12.13) tended to be older than the male subjects (mean age = 21.20; standard deviation = 10.08) (*p* = 0.088; mean rank female 107.34 and male 93.83). The age spread of the female patients was wider (Table 1).

**Table 1.** Comparison of the study participants with regard to sex distribution, age range, presence of parafunctional habits, and occlusion relationships, with prevalence data N (%).

	Study population N = 200 (%)	Male N = 98 (%)	Female N = 102 (%)
Age (yr) (25th, 50th, 75th percentile)	22.85, SD: 11.26 (13.5; 18.8; 31.2)	21.20, SD: 10.08 (13.0; 18.0; 28.3)	24.42, SD: 12.13 (14.0; 19.5; 34.0)
Angle Class I malocclusion	85 (42.5%)	39 (39.8%)	36 (35.3%)
Angle Class II malocclusion	112 (56.0%)	52 (53.1%)	58 (56.9%)
Angle Class III malocclusion	13 (6.5%)	6 (6.1%)	7 (6.9%)
Deep bite (Ob ≥3.5 mm)	80 (40.0%)	39 (39.8%)	41 (40.2%)
Fontal open bite (Ob ≤0.0 mm)	9 (4.5%)	5 (5.1%)	4 (3.9%)
Lateral crossbite	29 (14.5%)	16 (16.3%)	13 (12.7%)
Lateral open bite	17 (8.5%)	11 (11.2%)	6 (5.9%)
Parafunction, abnormal Attrition	160 (80.0%)	78 (79.6%)	82 (80.4%)

*SD: standard deviation; Ob: overbite.*

A total of 80.0% of the study participants had attrition, balance and/or hyperbalance contacts. The data indicates that 42.5% of cases exhibited neutral occlusion, 56.0% demonstrated distal occlusion, and 6.5% showed mesial occlusion (Table 1).

### 3.1 Compensated findings in asymptomatic patients

Compensated findings of all five DC/TMD leading symptoms were detectable with different prevalence values with all four TMD screening tests: 0.0%–15.0% compensated arthralgia, 14.5%–20.5% DD, 34.5%–49.5% compensated myogenic pain, 0.5%–1.0% DD w/o R, and 0.0%–0.5% compensated DJD (Table 2).

**Table 2.** Comparison of the four screening tests and the Diagnostic Criteria for Temporomandibular Disorders concerning their prevalence N (%) and *p*value per diagnosis.

Test	Arthralgia N (%)	DD N (%)	DD w/o R N (%)	DJD N (%)	Myo N (%)
PMSA	30 (15.0%)	40 (20.0%)	2 (1.0%)	1 (0.5%)	99 (49.5%)
PSSS	30 (15.0%)	40 (20.0%)	2 (1.0%)	1 (0.5%)	99 (49.5%)
CMD-Screening	23 (11.5%)	41 (20.5%)	1 (0.5%)	1 (0.5%)	69 (34.5%)
CMD-Short Finding	0 (0.0%)	29 (14.5%)	1 (0.5%)	0 (0.0%)	92 (46.0%)
DC/TMD unadapted	0 (0.0%)	23 (11.5%)	1 (0.5%)	0 (0.0%)	0 (0.0%)
DC/TMD adapted	25 (12.5%)	-	-	1 (0.5%)	82 (41.0%)^a^
DC/TMD adapted					101 (50.5%)^b^
*p*-value Cochran Q test	<0.001	<0.001	0.392	0.392	<0.001

*Myo: myogenic pain; DD: disc displacement with reduction; DD w/o R: disc displacement without reduction with limited opening; DJD: degenerative joint disease; ^a^: prevalence N (%) with exclusion digastric muscle; ^b^: prevalence N (%) with inclusion digastric muscle; PMSA: Preventive Manual Structural Analysis; PSSS: Preventive Structural Stress Screening; DC/TMD: Diagnostic Criteria for Temporomandibular Disorders; CMD: craniomandibular disorders.*

For compensated DJD (*p* = 0.392) and DD w/o R (*p* = 0.392), no significant differences between the prevalence screenings could be detected using the Cochran Q test (Table 2). The prevalence of the four tests differed significantly regarding compensated arthralgia (*p* < 0.001), compensated myogenic pain (*p* < 0.001), and the onset of DD (*p* < 0.001; Table 2). For these three DC/TMD diagnoses, pairwise comparisons of the screenings were performed using McNemar tests.

There were no significant differences between the PMSA and PSSS regarding compensated arthralgia and myogenic pain or DD (*p* = 1.000 in each case). In compensated arthralgia, the CMD-Short Finding differed significantly from the CMD-Screening (*p* < 0.001), PMSA (*p* < 0.001), and PSSS (*p* < 0.001). Furthermore, a significant difference was found between the CMD-Short Finding and all other screening tests (*p* < 0.001) in the case of DD. Concerning compensated myogenic pain, the CMD-Short Finding found no significant difference from the PMSA (*p* = 0.490) and PSSS (*p* = 0.490), but a significant difference from the CMD-Screening (*p* < 0.001). The CMD-Screening demonstrated no significant differences between the PMSA and PSSS regarding compensated arthralgia (*p* = 0.528 each) and DD (*p* = 1.000 each). For compensated myogenic pain, CMD-Screening differed significantly from the PMSA and PSSS (*p* < 0.001 each).

### 3.2 Evaluation of the four TMD screening tests using sensitivity and specificity

#### 3.2.1 Decompensated findings

When using the unadapted DC/TMD diagnostic algorithm, zero values occurred for arthralgia, muscle, and degenerative joint findings. Sensitivity, PPV, and *p*-value could not be calculated for these diagnoses. In each case, the observed study prevalence was 0.00%, falling below the corresponding prevalences. The NPV of the four tests decreased significantly for these three diagnoses with a change to the reference prevalence. For arthralgia, the specificity values of the individual screening tests were high and the NPV very high (Table 3, Ref. [29, 31, 32]). For decompensated myogenic pain, the specificity values were insufficient for all four screenings and the NPV with study prevalence was very high (Table 4, Ref. [1, 29, 30, 31]). For the degenerative joint findings, the specificity values of all screening tests were insufficiently high (Table 5, Ref. [29, 31, 33]). For these three DC/TMD diagnoses, positive findings were rather unlikely to be detected with DC/TMD compared to PMSA (OR_DC/TMD_ <0.001, OR_PMSA_ = 1.000).

**Table 3.** Comparison of the screening tests for arthralgia and the Diagnostic Criteria for Temporomandibular Disorders with adapted and unadapted diagnostic algorithm using *p*-values, sensitivity, specificity, positive predictive value, negative predictive value, and confidence interval 95% CI.

Test	Sensitivity % (CI)	Specificity % (CI)	Positive predictive value % (CI)		Negative predictive value % (CI)		*p*-value
DC/TMD with diagnostic algorithm			Prevalence 0.00%^a^	Prevalence 6.18%^b^	Prevalence 0.00%^a^	Prevalence 6.18%^b^	McNemar
PMSA	/	85.00 (79.28, 89.65)	0.00	0.00	100.00	92.81 (87.83, 96.20)	/
PSSS	/	85.00 (79.28, 89.65)	0.00	0.00	100.00	92.81 (87.83, 96.20)	/
CMD-Short Finding	/	100.00 (98.17, 100.00)	0.00	/	100.00	93.82 (89.53, 96.73)	/
CMD-Screening	0.00 (0.00, 84.19)	88.50 (83.25, 92.57)	0.00	0.00	98.88 (98.83, 98.94)	93.07 (88.28, 96.34)	/
DC/TMD without diagnostic algorithm			Prevalence 12.50%^a^	Prevalence 6.18%^b^	Prevalence 12.50%^a^	Prevalence 6.18%^b^	
PMSA	96.00 (79.65, 99.90)	96.57 (92.69, 98.73)	80.00 (64.47, 89.81)	64.84 (45.55, 80.25)	99.41 (96.12, 99.91)	99.73 (98.17, 99.96)	0.125
PSSS	96.00 (79.65, 99.90)	96.57 (92.69, 98.73)	80.00 (64.47, 89.81)	64.84 (45.55, 80.25)	99.41 (96.12, 99.91)	99.73 (98.17, 99.96)	0.125
CMD-Short Finding	0.00 (0.00, 13.72)	100.00 (97.91, 100.00)	/	/	87.50 (87.50, 87.50)	93.82 (93.82, 93.82)	/
CMD-Screening	92.00 (73.97, 99.02)	100.00 (97.91, 100.00)	100.00	100.00 (85.18, 100.00)	98.87 (95.86, 99.70)	99.48 (98.05, 99.86)	0.500

*PPV and NPV were presented with the study prevalence a and the reference prevalence b [31, 32] with age-specific weighting [29]. PMSA: Preventive Manual Structural Analysis; PSSS: Preventive Structural Stress Screening; CMD: craniomandibular disorders; DC/TMD: Diagnostic Criteria for Temporomandibular Disorders; CI: confidence intervals.*

**Table 4.** Comparison of the screening tests for myogenic pain and the Diagnostic Criteria for Temporomandibular Disorders with adapted and unadapted diagnostic algorithm with and without including the digastric muscle using *p*-values, sensitivity, specificity, positive predictive value, negative predictive value, and confidence interval 95% CI.

Test	Sensitivity % (CI)	Specificity % (CI)	Positive predictive value % (CI)		Negative predictive value % (CI)		*p*-value
DC/TMD with diagnostic algorithm			Prevalence 0.00%^a^	Prevalence 12.53%^b^	Prevalence 0.00%^a^	Prevalence 12.53%^b^	McNemar
PMSA	/	50.50 (43.36, 57.63)	0.00	0.00	100.00	77.91 (68.56, 85.56)	/
PSSS	/	50.50 (43.36, 57.63)	0.00	0.00	100.00	77.91 (68.56, 85.56)	/
CMD-Short Finding	/	54.00 (46.83, 61.05)	0.00	0.00	100.00	79.04 (70.15, 86.28)	/
CMD-Screening	/	65.50 (58.47, 72.06)	0.00	0.00	100.00 (97.22, 100.00)	82.06 (74.40, 88.21)	/
DC/TMD without diagnostic algorithm			Prevalence 41.00%^a^	Prevalence 12.53%^b^	Prevalence 41.00%^a^	Prevalence 12.53%^b^	
PMSA	97.56 (91.47, 99.70)	83.90 (76.00, 90.02)	80.81 (73.58, 86.42)	46.46 (36.47, 56.74)	98.02 (92.63, 99.49)	99.59 (98.39, 99.89)	<0.001
PSSS	97.56 (91.47, 99.70)	83.90 (76.00, 90.02)	80.81 (73.58, 86.42)	46.46 (36.47, 56.74)	98.02 (92.63, 99.49)	99.59 (98.39, 99.89)	<0.001
CMD-Short Finding	91.46 (83.20, 96.50)	85.59 (77.94, 91.38)	81.52 (73.88, 87.31)	47.62 (36.82, 58.65)	93.52 (87.62, 96.71)	98.59 (97.17, 99.30)	0.064
CMD-Screening	84.15 (74.42, 91.28)	100.00 (96.92, 100.00)	100.00	100.00 (94.79, 100.00)	90.08 (84.65, 93.73)	97.78 (96.40, 98.64)	<0.001
DC/TMD without diagnostic algorithm with digastric muscle			Prevalence 50.50%^a^	Prevalence 12.53%^b^	Prevalence 50.50%^a^	Prevalence 12.53%^b^	
PMSA	98.02 (93.03, 99.76)	100.00 (96.34, 100.00)	100.00	100.00 (96.34, 100.00)	98.02 (92.62, 99.49)	99.72 (98.89, 99.93)	0.500
PSSS	98.02 (93.03, 99.76)	100.00 (96.34, 100.00)	100.00	100.00 (96.34, 100.00)	98.02 (92.62, 99.49)	99.72 (98.89, 99.93)	0.500
CMD-Short Finding	91.09 (83.76, 95.84)	100.00 (96.34, 100.00)	100.00	100.00 (96.07, 100.00)	91.67 (85.50, 95.35)	98.74 (97.67, 99.32)	0.004
CMD-Screening	68.32 (58.31, 77.22)	100.00 (96.34, 100.00)	100.00	100.00 (94.79, 100.00)	75.57 (69.91, 80.47)	95.66 (94.30, 96.70)	<0.001

*PPV and NPV were presented with the study prevalence a and the reference prevalence b [1, 30, 31] with age-specific weighting [29]. PMSA: Preventive Manual Structural Analysis; PSSS: Preventive Structural Stress Screening; CMD: craniomandibular disorders; DC/TMD: Diagnostic Criteria for Temporomandibular Disorders; CI: confidence intervals.*

**Table 5.** Comparison of the screening tests for degenerative joint disease and the Diagnostic Criteria for Temporomandibular Disorders with adapted and unadapted diagnostic algorithm using *p*-values, sensitivity, specificity, positive predictive value, negative predictive value, and confidence interval 95% CI.

Test	Sensitivity % (CI)	Specificity % (CI)	Positive predictive value % (CI)		Negative predictive value % (CI)		*p*-value
DC/TMD with diagnostic algorithm			Prevalence 0.00%^a^	Prevalence 27.94%^b^	Prevalence 0.00%^a^	Prevalence 27.94%^b^	McNemar
PMSA	/	99.50 (97.25, 99.99)	0.00	0.00	100.00	71.96 (65.17, 78.08)	/
PSSS	/	99.50 (97.25, 99.99)	0.00	0.00	100.00	71.96 (65.17, 78.08)	/
CMD-Short Finding	/	100.00 (98.17, 100.00)	/	/	100.00	72.06 (65.29, 78.16)	/
CMD-Screening	/	99.50 (97.25, 99.99)	0.00	0.00	100.00	71.96 (65.17, 78.08)	/
DC/TMD without diagnostic algorithm			Prevalence 0.50%^a^	Prevalence 27.94%^b^	Prevalence 0.50%^a^	Prevalence 27.94%^b^	
PMSA	100.00 (2.50, 100.00)	100.00 (98.16, 100.00)	100.00	100.00 (2.50, 100.00)	100.00	100.00 (98.16, 100.00)	1.000
PSSS	100.00 (2.50, 100.00)	100.00 (98.16, 100.00)	100.00	100.00 (2.50, 100.00)	100.00	100.00 (98.16, 100.00)	1.000
CMD-Short Finding	0.00 (0.00, 97.50)	100.00 (98.16, 100.00)	/	/	99.50 (99.50, 99.50)	72.06 (72.06, 72.06)	/
CMD-Screening	100.00 (2.50, 100.00)	100.00 (98.16, 100.00)	100.00	100.00 (2.50, 100.00)	100.00	100.00 (98.16, 100.00)	1.000

*PPV and NPV were presented with the study prevalence a and the reference prevalence b [31, 33] with age-specific weighting [29]. PMSA: Preventive Manual Structural Analysis; PSSS: Preventive Structural Stress Screening; CMD: craniomandibular disorders; DC/TMD: Diagnostic Criteria for Temporomandibular Disorders; CI: confidence intervals.*

#### 3.2.2 Compensated arthralgia findings using the adapted DC/TMD diagnostic algorithm

The PMSA, PSSS, and CMD-Screening fulfilled the requirement of at least a 70% sensitivity [34]. All screenings achieved a specificity of 95% [34]. The CMD-Short Finding showed a sensitivity of 0.0% [0.00; 97.50], and a *p*-value could not be calculated. The PMSA (*p* = 0.125), PSSS (*p* = 0.125), and CMD-Screening (*p* = 0.500) did not differ significantly from the DC/TMD. Considering the right/left correlation, the PMSA also did not differ significantly from the DC/TMD (*p* = 0.063, OR_PMSA_ = 1.000, OR_DC/TMD_ = 0.860) (Table 3).

The study prevalence (12.50%) for compensated arthralgia findings was 2.02 times higher than the reference prevalence (6.18%) for decompensated arthralgia findings. The PPVs of the PMSA and the PSSS decreased compared to the reference prevalence. The NPVs of the four screenings increased slightly with the reference prevalence. The different prevalences did not influence the PPV of the CMD-Screening.

#### 3.2.3 Compensated myogenic pain findings without the digastric muscle using the adapted DC/TMD diagnostic algorithm

Unlike other tests, the CMD-Screening achieved a 95% specificity [34]. All four screening tests exceeded the required sensitivity of 70% [34]. The PMSA (*p* < 0.001), PSSS (*p* < 0.001), and CMD-Screening (*p* < 0.001) differed significantly from DC/TMD. The CMD-Short Finding demonstrated no significant difference from DC/TMD (*p* = 0.064). The PMSA also differed significantly from the DC/TMD (*p* = 0.003, OR_PMSA_ = 1.000, OR_DC/TMD_ = 0.808), considering the right/left correlation (Table 4).

The study prevalence with compensated muscle findings (41.0%) was 3.33 times higher than the reference prevalence with decompensated muscle findings (12.53%). The PPVs of the PMSA, PSSS, and CMD-Short Finding were lower with the reference prevalence than with study prevalence. The opposite was true for the NPV of the four tests. The PPV of the CMD-Screening remained unchanged with the change in prevalence.

#### 3.2.4 Compensated myogenic pain findings with the digastric muscle using the adapted DC/TMD diagnostic algorithm

Except for the CMD-Screening, all screening tests achieved the required 70% sensitivity [34], and all four screening tests reached the required 95% specificity [34]. The CMD-Screening (*p* < 0.001) and CMD-Short Finding (*p* = 0.004) differed significantly from the DC/TMD. The PMSA (*p *= 0.500) and PSSS (*p* = 0.500) displayed no significant difference from the DC/TMD. There was also no significant difference between PMSA and DC/TMD (*p* = 0.055, OR_PMSA_ = 1.000; OR_DC/TMD_ = 1.064), considering the right/left correlation (Table 4).

The study prevalence (50.5%) was 4.10 times higher than the reference prevalence (12.53%) and higher than the study prevalence excluding the digastric muscle (41.0%). The lower the NPVs of the four screening tests, the more it increased with lower reference prevalence. The PPVs of the four tests did not change when the prevalences changed.

#### 3.2.5 Compensated DJD findings using the adapted DC/TMD diagnostic algorithm

Except for the CMD-Short Finding, all screenings achieved the required 70% sensitivity and the 95% specificity [34]. The PMSA, PSSS, and CMD-Screening did not differ significantly from the DC/TMD (*p* = 1.000 each). The sensitivity of the CMD-Short Finding was 0.0% [0.00; 97.50], and no *p*-value could be determined. Considering the right/left correlation, no *p*-value could be calculated, the OR for comparing the PMSA with the DC/TMD could be calculated (OR_PMSA_ = 1.000, OR_DC/TMD_ = 1.000) (Table 5).

The study prevalence (0.50%) was significantly lower than the reference prevalence (27.94%). The NPV of the CMD-Short Finding was lower with reference prevalence than with study prevalence. The NPV and PPV of the other tests did not change with the change in prevalence.

#### 3.2.6 DD w/o R using the unadapted DC/TMD diagnostic algorithm

All four screening tests exceeded the required 70% sensitivity and the 95% specificity [34] and did not differ significantly from the DC/TMD (*p* = 1.000 each). The side-specific comparison between the PMSA and DC/TMD was omitted (Table 6, Ref. [29, 31, 33]).

**Table 6.** Comparison of the screening tests for disc displacement without reduction with limited opening and the Diagnostic Criteria for Temporomandibular Disorders with unadapted diagnostic algorithm using *p*-values, sensitivity, specificity, positive predictive value, negative predictive value, and confidence interval 95% CI.

Test	Sensitivity % (CI)	Specificity % (CI)	Positive predictive value % (CI)		Negative predictive value % (CI)		*p*-value
DC/TMD with diagnostic algorithm			Prevalence 0.50%^a^	Prevalence 9.82%^b^	Prevalence 0.50%^a^	Prevalence 9.82%^b^	McNemar
PMSA	100.00 (2.50, 100.00)	99.50 (97.23, 99.99)	50.00 (12.40, 87.60)	95.59 (75.42, 99.35)	100.00	100.00 (98.15, 100.00)	1.000
PSSS	100.00 (2.50, 100.00)	99.50 (97.23, 99.99)	50.00 (12.40, 87.60)	95.59 (75.42, 99.35)	100.00	100.00 (98.15, 100.00)	1.000
CMD-Short Finding	100.00 (2.50, 100.00)	100.00 (98.16, 100.00)	100.00	100.00 (2.50, 100.00)	100.00	100.00 (98.16, 100.00)	1.000
CMD-Screening	100.00 (2.50, 100.00)	100.00 (98.16, 100.00)	100.00	100.00 (2.50, 100.00)	100.00	100.00 (98.16, 100.00)	1.000

*PPV and NPV were presented with the study prevalence a and the reference prevalence b [31, 33] with age-specific weighting [29]. PMSA: Preventive Manual Structural Analysis; PSSS: Preventive Structural Stress Screening; CMD: craniomandibular disorders; DC/TMD: Diagnostic Criteria for Temporomandibular Disorders; CI: confidence intervals.*

The study prevalence (0.50%) was lower than the reference prevalence (9.82%). The PPVs of the PMSA and the PSSS were higher with reference prevalence than with study prevalence. The PPVs of the CMD-Screening and the CMD-Short Finding, as well as the NPVs of all four tests, remained unchanged when the prevalences were changed.

#### 3.2.7 DD using the unadapted DC/TMD diagnostic algorithm

All four screening tests exceeded the required 70% sensitivity [34]. The CMD-Short Finding was the only test to achieve 95% specificity [34]. All four screening tests differed significantly from the DC/TMD (PMSA: *p* < 0.001, PSSS: *p* < 0.001, CMD-Screening: *p* < 0.001, and CMD-Short Finding: *p* = 0.031). The PMSA also differed significantly from the DC/TMD in the side-specific comparison (*p* < 0.001, OR_PMSA_ = 1.000, OR_DC/TMD_ = 0.436) (Table 7, Ref. [29, 31, 33]).

**Table 7.** Comparison of the screening tests for disc displacement with reduction and the Diagnostic Criteria for Temporomandibular Disorders with unadapted diagnostic algorithm using *p*-values, sensitivity, specificity, positive predictive value, negative predictive value, and confidence interval 95% CI.

Test	Sensitivity % (CI)	Specificity % (CI)	Positive predictive value % (CI)		Negative predictive value % (CI)		*p*-value
DC/TMD with diagnostic algorithm			Prevalence 11.50%^a^	Prevalence 24.18%^b^	Prevalence 11.50%^a^	Prevalence 24.18%^b^	McNemar
PMSA	100.00 (85.18, 100.00)	90.40 (85.07, 94.30)	57.50 (46.26, 68.01)	76.86 (67.88, 83.92)	100.00	100.00 (97.72, 100.00)	<0.001
PSSS	100.00 (85.18, 100.00)	90.40 (85.07, 94.30)	57.50 (46.26, 68.01)	76.86 (67.88, 83.92)	100.00	100.00 (97.72, 100.00)	<0.001
CMD-Short Finding	100.00 (85.18, 100.00)	96.61 (92.77, 98.75)	79.31 (63.58, 89.38)	90.39 (81.08, 95.38)	100.00	100.00	0.031
CMD-Screening	100.00 (85.18, 100.00)	89.83 (84.40, 93.86)	56.10 (45.20, 66.44)	75.82 (66.93, 82.93)	100.00	100.00 (97.71, 100.00)	<0.001

*PPV and NPV were presented with the study prevalence a and the reference prevalence b [31, 33] with age-specific weighting [29]. PMSA: Preventive Manual Structural Analysis; PSSS: Preventive Structural Stress Screening; CMD: craniomandibular disorders; DC/TMD: Diagnostic Criteria for Temporomandibular Disorders; CI: confidence intervals.*

The study prevalence of DD (11.50%) was 2.10 times lower than the reference prevalence (24.18%). The PPVs of the four screening tests were higher with reference prevalence than with study prevalence. The NPVs of the four tests remained unchanged when the prevalences were changed.

## 4. Discussion

### 4.1 Discussion of the results

Three working hypotheses were formulated:

(A) The screening tests differ in their diagnostic quality.

(B) The screening tests differ from the reference standard.

(C) The screening tests differ in terms of prevalence.

Some differences occurred between the screening tests and reference standard, which can be attributed to the absence of symptoms in the patients per the medical history.

Because the population was asymptomatic, the unadapted DC/TMD diagnostic algorithms produced only zero values for decompensated arthralgia, myogenic pain, and DJD. As there were no true positive cases, sensitivity, PPVs, and *p*-values could not be calculated for these three diagnoses. For decompensated myogenic pain, all tests exhibited insufficient specificity values and very high NPVs with study prevalence. Decompensated arthralgia also led to insufficient specificity and high NPVs. However, the CMD-Short Finding showed very high specificity but failed to detect any cases. For decompensated DJD, the screenings had very high specificity because the study prevalence was very low.

The adaptations of the diagnostic algorithms described above confirmed the compensated findings regarding arthralgia, DJD, and myogenic pain. The DD and DD w/o R were detectable with unadapted diagnostic algorithms because this did not require a positive medical history [22].

#### 4.1.1 Compensated arthralgia

The CMD-Short Finding does not include a specific parameter that is capable of detecting arthralgia findings. Its prevalence (0.0%) differed significantly from that of the other tests (*p* < 0.001). Due to the zero values, no sensitivity, PPV, or *p*-value (comparison with reference standard) could be specified. The present study found that PMSA and PSSS (with 15.0% each) detected a greater number of compensated findings by passive compression than DC/TMD (12.5%) and CMD-Screening (11.5%) with palpation. This resulted in higher false-positive rates and lower PPVs (at study prevalence: 80.00% [64.47–89.81], at reference prevalence: 64.84% [45.55–80.25]). The broad CI of the sensitivity (96.00% [79.65–99.90]) could be reduced by an increased number of cases.

PMSA (*p* = 0.125, side-specific *p* = 0.063), PSSS (*p* = 0.125), and CMD-Screening (*p* = 0.500) did not differ significantly from the reference standard and hypothesis B was rejected for these screening tools. They also did not differ from each other (PMSA *vs.* PSSS: *p* = 1.000; CMD-Screening *vs.* PMSA *p* = 0.528, CMD-Screening *vs.* PSSS *p* = 0.528).

Hypotheses A and C were retained exclusively for the CMD-Short Finding, as it was the only one that didn’t meet the minimum diagnostic requirements and differed significantly from the other screening tools in terms of prevalence.

#### 4.1.2 Compensated myogenic pain

For compensated myogenic pain, when using the DC/TMD without including the digastric muscle, the CMD-Screening represented the sole test that attained the required 95% specificity (100.00% [96.92–100.00]) [34]. This is attributable to the fact that the test exclusively examined the masseter muscle pars superficialis and temporalis muscle pars anterior/media/posterior. The prevalence of CMD-Screening was significantly found to be the lowest of the four screening tools (*p* < 0.001). The PMSA and PSSS exhibited the lowest specificity levels and differed significantly from the reference standard (each 83.90% [76.00–90.02], *p* < 0.001, PMSA side-specific *p* = 0.003). This is attributable to the fact that both tests palpate the digastricus venter anterior and posterior, in addition to the masseter and temporalis muscles and the examination of passive mouth opening. The CMD-Short Finding also demonstrated insufficient specificity yet exhibited no significant discrepancy from DC/TMD (85.59% [77.94–91.38], *p* = 0.064), as the digastricus pars posterior was the sole muscle examined, in conjunction with the masseter pars superficialis and temporalis pars anterior.

Hypotheses A, B and C were retained exclusively for the CMD-Screening, as it was the only one that met the minimum diagnostic requirements, showed no significant difference to the reference standard and differed significantly from the other screening tools in terms of prevalence.

When the reference standard included the digastric muscle anterior/posterior bellies, the opposite was true. The CMD-Screening was the only test that did not achieve the required 70% sensitivity [34] and, like the CMD-Short Finding (*p* = 0.004), differed significantly from the DC/TMD (*p* < 0.001). The PMSA and PSSS did not differ significantly from the reference standard (*p* = 0.500, PMSA side-specific *p* = 0.055). The different palpation combinations used in the tests led to varying prevalence rates and explain this observation.

Hypotheses A and C were retained exclusively for the CMD-Screening, as it was the only one that met not the minimum diagnostic requirements and differed significantly from the other screening tools in terms of prevalence. Hypothesis B were retained only for CMD-Short Finding and CMD-Screening.

#### 4.1.3 Disc displacement

Concerning DD, the DC/TMD diagnostic algorithm required cracking noise to occur either during the opening and closing of the mouth or in combination with excursive movements [22]. In the CMD-Screening, the clicking noise was counted during active movements regardless of the combination [14, 22]. In the PMSA and the PSSS, the clicking noise was detected using dynamic translation [19, 20]. The CMD-Short Finding analyzed only cracking noise during mouth opening or closing [16, 17] and, therefore, had a higher specificity (96.61% [92.77–98.75]) and a significantly lower prevalence (14.5%, *p* < 0.001) than the other tests.

Hypotheses A and C were retained exclusively for the CMD-Short Finding, as it was the only one that met the minimum diagnostic requirements and differed significantly from the other screening tools in terms of prevalence. In conclusion, clicking noise is more common in asymptomatic individuals during lateral and protrusive movements than in mouth opening or closing.

All screening tools differed significantly from the DC/TMD (PMSA *p* < 0.001, PMSA side-specific *p* < 0.001, PSSS *p* < 0.001, CMD-Screening *p* < 0.001, CMD-Short Finding *p* = 0.031). Hypothesis B were retained for all screening tools.

#### 4.1.4 Compensated degenerative joint disease

The CMD-Short Finding (0.0%) detected no compensated degenerative joint findings, unlike the other tests. Consequently, sensitivity, PPV, and *p*-value could not be calculated. This test assessed joint sounds only during mouth opening/closing, not during excursive movements like the other screening tools and DC/TMD. In one case, crepitus occurred during protrusion and was detected by both the reference standard (0.5%) and the other TMD screening tests (0.5%). Due to the extremely low prevalence, all tests indicated a wide CI for sensitivity and the results should be interpreted with caution. All screening tests, except for the CMD-Short Finding, met the required thresholds of at least a 70% sensitivity and a 95% specificity and had no significant difference from DC/TMD (each *p* = 1.000). Therefore, hypotheses A and B were rejected for all tests except the CMD-Short Finding.

The screening tools did not differ significantly in prevalence (*p* = 0.392), so hypothesis C was also rejected (for all screening tools).

#### 4.1.5 Disc displacement without reduction with limited opening

In DD w/o R, no significant differences were detectable between the screenings (*p* = 0.392) and the DC/TMD (*p* = 1.000) despite the different thresholds for RMO (PMSA and PSSS <43 mm, DC/TMD and CMD-Screening <40 mm, CMD-Short Finding <38 mm). The PMSA and PSSS exhibited a false positive value, resulting in a lower PPV (each 50.00% [12.40–87.60] at study prevalence; each 95.59% [75.42–99.35] at reference prevalence). All tests showed wide CIs for sensitivity due to the low study prevalence, and the results should be interpreted with caution.

Hypotheses A, B and C were rejected for all tests, as they all met the minimum diagnostic requirements, showed no significant differences from the reference standard, and did not differ significantly in prevalence.

The low prevalence of compensated DJD and DD w/o R reveals that such findings are rarely unnoticed and play a subordinate role in patients with no history of symptoms.

### 4.2 Summation of the comparison

The CMD-Short Finding is notable for its good comprehensibility. No structured training is required [16]. The CMD-Short Finding can detect hidden muscle findings involving the mouth opening, DD, and DD w/o R. When digastric muscle is included in the assessment, the CMD-Short Finding is suitable for detecting hidden muscle findings. Furthermore, this test can detect DD and DD w/o R. This outcome was achieved despite the study design, which enabled parameters to be individually considered without summation. With regard to sensitivity, PPV, specificity, and prevalence, this test is inadequate for detecting compensated arthralgia and DJD. A subsequent study employing summed parameters for these two diagnoses would be interesting (Fig. 9).

**Fig. 9.** Flowchart for the comparison of the four screening tests corresponding to the five diagnoses and the achieved targets of a 70% sensitivity and a 95% specificity s, no significant difference to the Diagnostic Criteria for Temporomandibular Disorders p and a recommendation based on prevalence n. Created by Maria Ertl. DD: disc displacement with reduction; DD w/o R: disc displacement without reduction with limited opening; DJD: degenerative joint disease; PMSA: Preventive Manual Structural Analysis; PSSS: Preventive Structural Stress Screening; CMD: craniomandibular disorders; DC/TMD: Diagnostic Criteria for Temporomandibular Disorders. –: target not achieved; √: target achieved.

The CMD-Screening allows a certain degree of freedom in the examination. However, it is recommended that the DC/TMD guidelines be followed [14]. In terms of sensitivity and specificity, this test is well suited for detecting compensated arthralgias, compensated DJD, and DD w/o R. This test is unsuitable for DD and compensated myogenic pain, considering the digastric muscle. With regard to prevalence, the CMD-Screening is also effective in identifying DD. However, questions about discomfort and pain may be of limited value in asymptomatic patients as participants denied such symptoms despite having compensated findings (Fig. 9).

The PMSA and PSSS are based on manual examination techniques [19] and require some experience. Compared to the PMSA, the PSSS finding sheet has a more straightforward structure. Both tests provide the same results, although the PSSS is a condensed version of the PMSA. Therefore, the PSSS may be a suitable alternative to the PMSA, and further studies of symptomatic patients should be conducted. Concerning sensitivity, specificity, and including the digastric muscle for myogenic pain, the PMSA and PSSS are suitable for detecting four of the five DC/TMD diagnoses. The required 95% specificity [34] was not achieved for DD. Regarding prevalence, both tests are well suited for detecting all five DC/TMD diagnoses. Only the CMD-Screening displayed a slightly higher prevalence of DD without significant differences from PMSA and PSSS (*p* = 1.000) (Fig. 9).

### 4.3 The necessity of TMD screening tools

This study confirmed that asymptomatic patients may exhibit compensated masticatory findings, underscoring the relevance of TMD screening tests. The statement that screenings are unnecessary because symptomatic individuals would seek dental care [35, 36] was not confirmed in this study. Asymptomatic patients were unaware of their compensated findings. Furthermore, according to Bumann *et al*. [3], compensated findings can manifest as decompensated findings depending on the environmental influences and the body’s ability to adapt. Although the reported prevalences are not always therapeutically relevant, the cases are essential for documentation in the patient file. An examination of the stomatognathic system is indicated before each restorative and orthodontic treatment [6]. Disregarding this procedure constitutes malpractice in Germany. It is important not only to conduct a functional examination but also to document it [4, 5]. In this respect, fixed scheme TMD screening with basic documentation of findings is helpful.

### 4.4 The reference standard instead of the gold standard

In addition to magnetic resonance imaging and computed tomography scans, finding suitable gold standards in the specialized field of TMD to differentiate between “sick” and “healthy” patients is challenging [18]. For ethical reasons, no histological examinations can be performed on asymptomatic patients. Furthermore, there are no justifiable indications for X-rays or magnetic resonance imaging scans. Otherwise, there would be a breach of the Radiation Protection Act [37], and unnecessary costs would burden the healthcare system.

The DC/TMD is an internationally recognized examination method for symptomatic patients. Because of its reliability and validity, it is well suited for scientific work [21, 23, 24]. However, some authors argue that despite improvements over its predecessor (the RDC/TMD), there is insufficient evidence that the DC/TMD is suitable for research purposes [25]. A single question about facial pain can positively predict a TMD [38]. However, this study demonstrated that such questions are inappropriate in patients with no history of symptoms. Thus, despite the absence of reported pain, compensated findings were detectable only through the adapted diagnostic algorithms.

The results should be considered because no applicable gold standard [18] currently exists for asymptomatic patients. If the diagnostic adjustments are unjustified, then positive findings obtained using the DC/TMD Clinical Examination Protocol and screening tests can be described as false positives for arthralgia, myogenic pain, and DJD. But if the adjustments to the DC/TMD diagnostic algorithms are justified, positive findings obtained using the DC/TMD Clinical Examination Protocol and screening tests can be described as compensated findings. Furthermore, some screening tests, such as the PMSA, demonstrated a significantly higher prevalence for compensated findings than the DC/TMD (side-specific comparison: arthralgia: *p* = 0.063, DD: *p* < 0.001). The DC/TMD is an internationally accepted standard for symptomatic patients; however, this study design reveals that this may not be the best reference standard for asymptomatic patients. Further studies are needed to determine whether the PMSA would be a better reference standard.

### 4.5 Study limitations

Patients presenting with symptoms were intentionally excluded from the study population because it was deemed essential to align the study cohort with the profile of patients attending the dental practice who reported no symptoms. Moreover, DC/TMD was selected as the reference standard given that, as noted, no gold standard is currently available for asymptomatic patients. Adapting the DC/TMD algorithms for arthralgia, DJD, and myogenic pain to allow result interpretation may be considered a study limitation.

Moreover, side-specific and qualitative analyses were not conducted because the four TMD screening tests exhibited significant structural differences, precluding a meaningful comparison. Furthermore, ordinal parameters were summarized as nominal parameters. The prevalences were relatively low in some cases, necessitating larger sample sizes to yield meaningful results. Consequently, the study findings should be interpreted with caution. Nevertheless, this study provides a foundation for further research.

Given the very low prevalence of the condition, as is the case with DJD or DD w/o R, even a single or two correctly positive cases can result in a sensitivity of 100% purely by chance. Binary evaluation is prone to overfitting, potentially leading to a misleading perception of test performance. The lower the prevalence, the wider the confidence interval. Seemingly perfect sensitivity values are statistically unreliable when derived from only a few true-positive cases. The PPV depends strongly on the prevalence, as the results show. Thus, even with high sensitivity and specificity, the PPV may remain low. At this juncture, the NPV should be favoured in clinical decision-making. The results of the study should be evaluated in light of these critical aspects, whilst acknowledging the limitations of the study.

The DC/TMD diagnostic algorithm stipulates that the presence of decompensated arthralgia and muscle findings is contingent upon the existence of both anamnestic pain and suitable positive known examination parameters [21]. Furthermore, the diagnosis of decompensated DJD could only be made if the examiners also heard the grinding noises [21]. In this study, subjects with no history of symptoms were explicitly examined, which is why no previously known grinding sounds noises and Familiar Pain could be confirmed by the examiner. Consequently, no decompensated findings for these three diagnoses were identifiable using DC/TMD. As previously outlined in the methodology, the DC/TMD diagnostic algorithm [22] was adapted for these three leading symptoms to enable the analysis of the data. Consequently, compensated findings could be identified in anamnestically asymptomatic individuals. Critically speaking, this approach deviates from the original DC/TMD reference standard. Conversely, a comparison of the screening tests would not have been feasible without a reference standard and without its adjustments.

It is important to note that significant variations exist among screening tests with regard to parameters, scale levels, and evaluation. In order to ensure comparability, the test parameters were assigned to the DC/TMD diagnoses. In some cases, ordinal scale levels were simplified to nominal scale levels. As only DC/TMD and PMSA were provided for side-separated examinations, the screening test comparison was not side-specific. To address this deficit, the PMSA was additionally evaluated side-specifically. The conscious acceptance of the concomitant loss of information and the limited comparability is evidenced.

### 4.6 Confirmation of quality

The participants were able to complete the questionnaires at home. The absence of double blinding constitutes a critical flaw, thereby compromising full objectivity. In order to compensate for this disadvantage, the participants were anonymized prior to the evaluation of the data.

To maintain examiner focus, the TMD screening tests were carried out in an alternating sequence. Subsequent to this, the DC/TMD was conducted in order to circumvent any potential bias on the part of the examiner. Furthermore, validated tests such as the DC/TMD [17], the CMD-Short Finding [15, 16, 17, 18], the CMD-Screening [14], and established tests such as the PMSA [19] and the PSSS [19, 20] were used.

The occurrence of random errors was mitigated through the implementation of standardized diagnostic forms and the repetition of measurements during active movements.

Systematic errors were avoided through meticulous measurements accurate to the millimetre, calibration of the palpation pressures, examinations under optimal lighting conditions, without time constraints and on a dentist’s chair. Furthermore, only reproducible findings were evaluated. At the time the examination was conducted, the examiner had accrued eight years of professional experience and in-depth knowledge in the field of functional diagnostics and therapy.

Although minor measurement deviations are inevitable [39] the points previously mentioned have been shown to reduce measurement errors, thereby ensuring the reliability and validity of the study.

## 5. Conclusions

All four screening tests detected a high degree of compensated findings in patients with no history of symptoms. Further research is needed to determine whether these findings have therapeutic relevance; however, the necessity of TMD screening tests was clearly demonstrated.

Based on prevalence, a recommendation can be made for the PMSA and PSSS for all five DC/TMD diagnoses. The CMD-Screening is suitable for all DC/TMD diagnoses except for compensated muscle findings. The CMD-Short Finding is suitable for all diagnoses except compensated arthralgia and compensated DJD. Not all tests consistently achieved the required 70% sensitivity and the 95% specificity, and there were significant differences from the DC/TMD. Questions about pain, as applied in the DC/TMD Symptom Questionnaire and CMD-Screening, are unsuitable for detecting compensated findings. However, the results should be considered in the context that no gold standard for asymptomatic patients currently exists, and that the prevalence of the study was very low in some cases. Hence, further studies with higher case numbers based on this study are needed. Future studies analyzing parameter summation in the CMD-Short Finding may provide deeper insight into arthralgia and DJD detection.
